# Dupuytren's Contracture Cosegregation with Limb-Girdle Muscle Dystrophy

**DOI:** 10.1155/2013/254950

**Published:** 2013-08-19

**Authors:** Baiba Lace, Inna Inashkina, Ieva Micule, Inta Vasiljeva, Maruta Solvita Naudina, Jurgis Strautmanis, Janis Stavusis, Eriks Jankevics

**Affiliations:** ^1^Latvian Biomedical Research and Study Centre, Ratsupites Street 1, Riga 1067, Latvia; ^2^Children Clinical University Hospital, Medical Genetics Clinic, Riga 1004, Latvia; ^3^Pauls Stradins Clinical University Hospital, Riga 1002, Latvia; ^4^Children Clinical University Hospital, Neurology Clinic, Riga 1004, Latvia

## Abstract

Limb-girdle muscular dystrophies (LGMDs) is a heterogeneous group of muscular dystrophies that mostly affect the pelvic and shoulder girdle muscle groups. We report here a case of neuromuscular disease associated with Dupuytren's contracture, which has never been described before as cosegregating with an autosomal dominant type of inheritance. Dupuytren's contracture is a common disease, especially in Northern Europe. Comorbid conditions associated with Dupuytren's contracture are repetitive trauma to the hands, diabetes, and seizures, but it has never before been associated with neuromuscular disease. We hypothesize that patients may harbor mutations in genes with functions related to neuromuscular disease and Dupuytren's contracture development.

## 1. Introduction 

Limb-girdle muscular dystrophies (LGMDs) is a heterogeneous group of muscular dystrophies that mostly affect the pelvic and shoulder-girdle muscle groups. LGMD is rare with an overall incidence of 1 per 100 000 people [1]. LGMD is classified into two main groups: autosomal dominant (LGMD type 1) and autosomal recessive (LGMD type 2). The more common type is autosomal recessive with 16 genes or chromosomal loci identified to date [2–4]. The rarer form is autosomal dominant. Ten percent of all cases of LGMD belong to this group. So far, eight genes or chromosomal loci have been associated with the autosomal dominant form [5–7].

 Diagnosis of the different types of LGMD is a challenging process due to the many genes involved, unidentified genes at known chromosomal loci, and the lack of common mutations in noninbred populations.

We report here a case of neuromuscular disease and Dupuytren's contracture, which has never been described before.

 Dupuytren's contracture appears initially as a nodule containing highly contractile myofibroblasts. The myofibroblasts gradually develop into the cord, which leads to progressive fibrosis of the palmar fascia [8]. Myosin light chain activation is the result of a series of events, triggered by fibroblast and myofibroblast migration and contraction [9].

 Dupuytren's contracture is a common disease. It is present in 4% of North European descendants [10]. Co-morbid conditions with Dupuytren's contracture are a history of smoking, repetitive trauma to the hands, diabetes, and seizures [11]. 

## 2. Case Report

**Figure 1:**
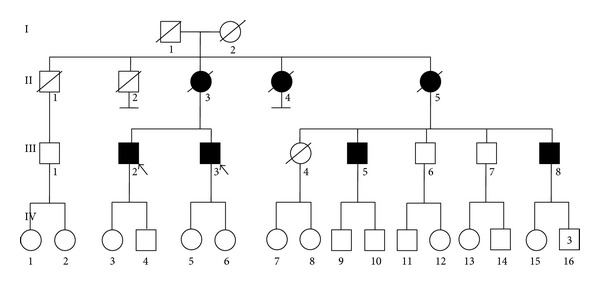
Family tree, black arrows indicate probands.

The patients were two brothers from a large family with multiple affected family members. See Figure 1.

III-2 Proband was a 62-year-old male, who complained of proximal muscle progressive weakness since the age of 50.

Proximal muscle weakness, with distal leg, and bilateral ankle dorsiflexor muscle weakness, and atrophy on physical examination were observed. Gowers symptom was positive. Neck flexor muscles showed mild weakness, but the facial muscles were normal. Dupuytren's contracture was in a palm.

Creatine kinase (CK) was mildly elevated at 527 IU/L. Electromyography revealed two processes concomitantly, chronic lumbosacral radiculopathy level L5-S1 and myopathic disease. Magnetic resonance imaging of the spine revealed lumbar degenerative disk disease and L4 vertebral retrolisthesis. Electrocardiography showed left ventricular hypertrophy. Limb-girdle muscle dystrophy (LGMD) was diagnosed. 

III-3 Proband was a 60-year-old male. His first complaints were muscle pain and gait disturbances, which started twelve years ago. He could not walk on his heels and after slow progression of the disease, he observed difficulties with climbing stairs.

**Figure 2:**
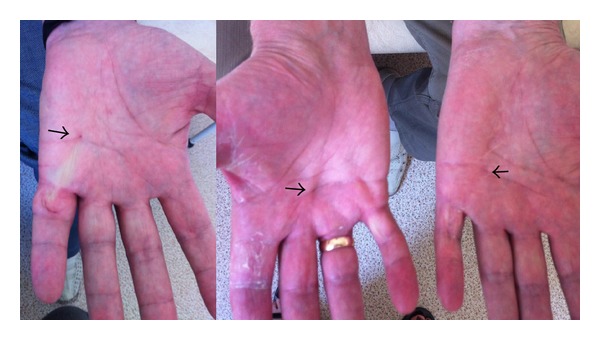
Hands of Probands III-2 and III-3. Arrows indicate contracture site. Distal muscle atrophy of hands.

Physical examination revealed shoulder-girdle muscle hypertrophy. Neck flexor muscles showed a mild weakness, but the facial muscles were normal. Scapular winging, proximal muscle weakness, with distal leg and bilateral ankle dorsiflexor muscle weakness and atrophy on physical examination were observed. Dupuytren's contractures were bilateral. See Figure 2.

All information about family members was obtained from the probands. Documentation about diagnosis was not available.

II-3 Person had proximal muscle weakness, which was more pronounced in the legs. He died at 92 years of age after a long period of immobility due to a hip fracture.

II-4 Person had proximal muscle weakness. He lost ambulation after twenty years.

II-5 Person was severely affected with muscle weakness. He lost ambulation at the age of 70.

III-5 Cousin had myopathy, proximal muscle weakness, and spinal deformity. He lost unaided walking at the age of 70.

III-8 Cousin had myopathy and proximal muscle weakness, but he is still ambulatory.

IV-3 Girl of 31 years of age, who had mild scapular winging and initial Dupuytren's contracture (or node) of the right palm.

## 3. DNA Diagnostics

Sequencing of the *CAV3* gene did not identify sequence variations in any of the analyzed family members (III-2, III-3, and IV-3). The most common mutations in the *LMNA* gene (1072G > A (Glu358Lys), 1357C > T (Arg453Trp) and 1718C > T (Ser573Leu)) were not observed. 

## 4. Discussion

 The risk of recurrence of Dupuytren's contracture recurrence for siblings is high (lambda ratio −2.9), indicating a strong genetic component, but it is a genetically heterogeneous disease. There have been familial reports about a mitochondrial and autosomal dominant type of inheritance [12]. Large families from Sweden showed isolated Dupuytren's contracture phenotype following autosomal dominant type of inheritance. Genome wide scan confirmed the highest LOD score in this family at chromosome 16q, but the gene was not identified [13]. 

 Molecular analysis employing large association studies and functional analysis have been performed. The results indicated that several pathways (Wnt, TGF*β*, Akt, Integrin, etc.) are involved in the pathogenesis of this disease. Also, the impairment of numerous genes and their functions underlies the development of Dupuytren's contracture; for instance, several collagen types and myosin (MYH8) [14] are dysregulated. We hypothesize that patients from this family may harbor mutations in genes with functions related to neuromuscular disease and Dupuytren's contracture development. To the best of our knowledge this is the first report of cosegregation of Dupuytren's contracture and neuromuscular disease. However, we could not exclude Dupuytren's contracture as an incidental finding in the family, but affected patients do not have any of the risk factors for the development of Dupuytren's contracture.
